# The implications of FASN in viral infection and related diseases: a promising target in antiviral therapies

**DOI:** 10.3389/fcimb.2026.1846176

**Published:** 2026-06-10

**Authors:** Yanfei Huang, Qiaoyu Xu, Lanlan Zheng, Huihua Zheng, Hongying Chen, Yilei Li, Shijie Ma

**Affiliations:** 1 Ministry of Education Key Laboratory for Animal Pathogens and Biosafety, College of Veterinary Medicine, Henan Agricultural University, Zhengzhou, Henan, China; 2 College of Veterinary Medicine, Key Laboratory for Animal-Derived Food Safety of Henan Province, Zhengzhou, Henan, China; 3 College of Animal Science and Technology and College of Veterinary Medicine, Zhejiang A&F University, Hangzhou, Zhejiang, China

**Keywords:** antiviral therapy, FASN, intracellular localization, post-translational modification, virology

## Abstract

Fatty acid synthase (FASN) is a crucial enzyme involved in fatty acid synthesis and has garnered increasing attention in virology, particularly concerning 13 zoonotic viruses, 16 human viruses, and 9 animal viruses. Recent advances in FASN research have uncovered several significant findings regarding viral replication. Specifically, FASN enhances BVDV replication by inhibiting the RIG-I/TBK-1 signaling pathway. Additionally, it modulates the palmitoylation of CHIKV nsP1, thereby increasing viral infectivity. Furthermore, FASN facilitates the proliferation of CSFV by promoting lipid droplet formation. It has also been reported that viral proteins can influence the mRNA levels, protein expression, and intracellular localization of FASN. This regulatory process involves various transcription factors and signaling pathways. Targeting FASN has been shown to impair fatty acid synthesis and reduce viral replication by pharmacological treatments or gene editing technologies. In this review, we will critically discuss recent studies on how the dysregulation of FASN contributes to viral infections and its potential as a promising target for antiviral therapy.

## Introduction

FASN is a key rate-limiting enzyme that is responsible for the *de novo* synthesis of the fatty acid palmitate (Gonzalez-Bohorquez et al., 2022; Wang et al., 2024; Xu et al., 2018). During this biological process, FASN catalyzes the conversion of malonyl CoA and acetyl CoA to palmitate in a nicotinamide adenine dinucleotide phosphate (NADPH)-dependent reaction (Vanauberg et al., 2023). Palmitate, a saturated free 16-carbon fatty acid, is subsequently converted to complex lipid molecules important for the elaboration of lipid rafts, protein modification and protein-protein signaling (Johari et al., 2024; Jones and Infante, 2015). Recently, it was shown that FASN is a pro-viral gene and inhibition of FASN effectively impairs the infectivity of various viruses (Aliyari et al., 2025; Chu et al., 2021; Liu et al., 2024, 2021; Mekhail et al., 2022; Williams et al., 2021; Zheng et al., 2024), including zoonotic, human, and animal viruses. With recent studies of the new development of FASN inhibitors (Chu et al., 2021; Polonio-Alcala et al., 2022; Xu et al., 2024), targeting FASN may be necessary for designing strategy for metabolically combating viral replication and virus-associated diseases.

FASN expression is upregulated in various malignancies (Li et al., 2024), tumor immune microenvironment (Xiao et al., 2024b) and viral propagation (Aliyari et al., 2022; Suroengrit et al., 2024). This review mainly focuses on the molecular mechanism of FASN expression and activity regulation in viral entry, viral production and emerging infectious diseases. Transcription factors, including sterol regulatory element-binding transcription factor 1 (SREBF1) (Shi et al., 2024), Yin Yang 1 (YY1) (Zheng et al., 2024), and forkhead box transcription factor O1 (FoxO1) (Bose et al., 2014) play a significant role in promoting the expression of FASN via binding to the promoter region of FASN. The majority of the evidence suggests deregulated expression of FASN has been linked to modulate the activity of phosphatidylinositol 3-kinase/protein kinase B (PI3K/AKT) (Liu et al., 2024), IFNα/β receptor/signal transducer and activator of transcription 1 (IFNAR/STAT1) (Aliyari et al., 2022), and AMP-activated protein kinase (AMPK)/SREBF1 signaling pathway (Meng et al., 2019).

**Figure 1:**
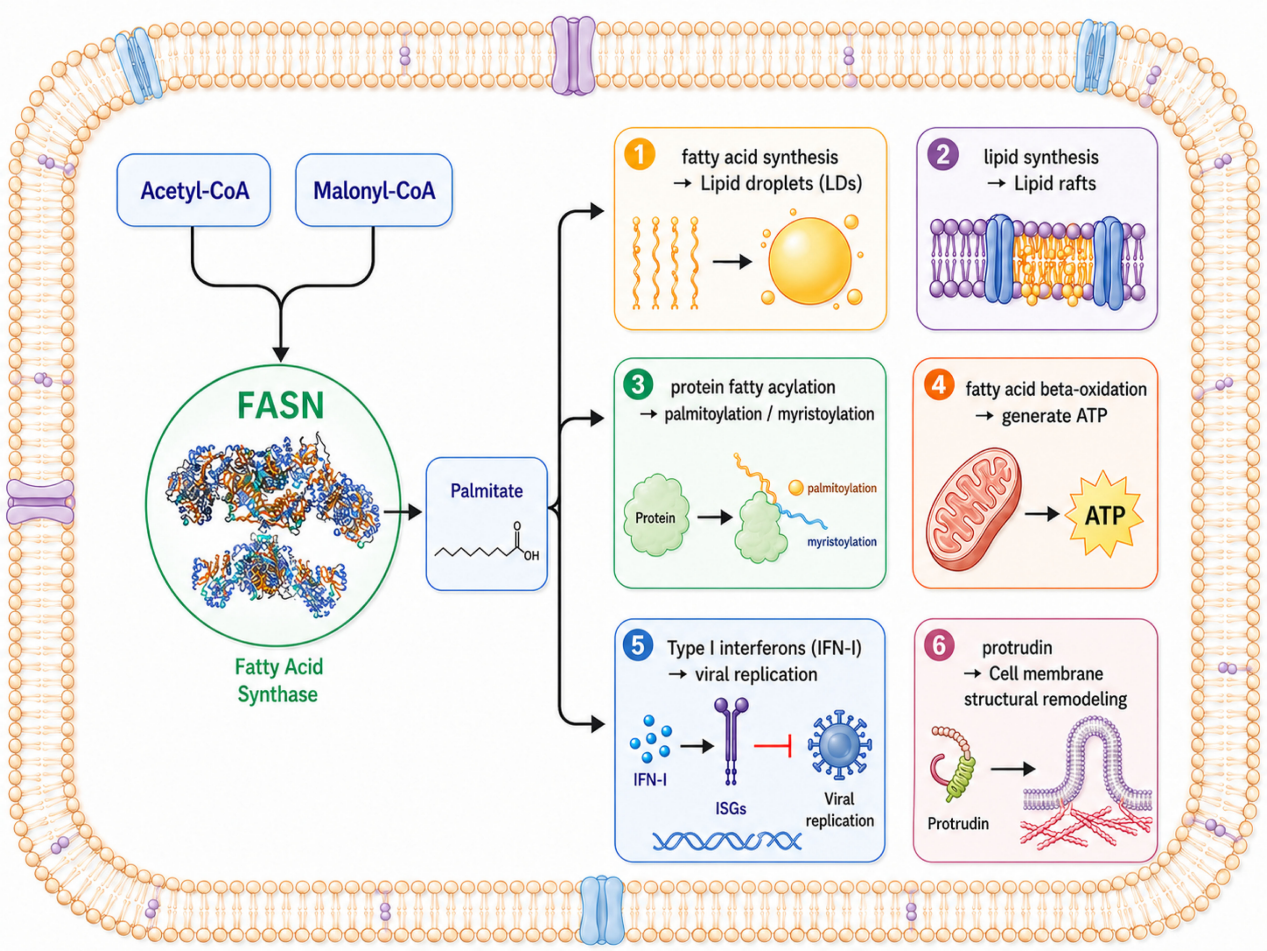
FASN-an integrated target in viral infection. FASN catalyzes acetyl-CoA and malonyl-CoA into palmitate. Palmitate is then converted into various fatty acids essential for lipid synthesis, fatty acid beta-oxidation, cell membrane structural remodeling, post-translation modification, and the host innate immune response.

From a biomolecular and pathological point of view, the function of FASN during virus growth was first identified in one research for inhibiting lytic Epstein-Barr virus (EBV) replication in patients (Li et al., 2004). Through the years, the increasing evidence suggests FASN inhibition restricts viral infection and related diseases by multiple mechanisms including altering the palmitoylation of some host and viral proteins (Karthigeyan et al., 2021; Williams et al., 2021; Zhang et al., 2019), fusion between viral and cellular membranes (Aliyari et al., 2022), ATP production (Liu et al., 2024), and interactions with other pro/anti-viral signaling pathways (Liu et al., 2024; Yu et al., 2020) (**Figure 1**). Furthermore, host lipid synthesis, especially fatty acid synthesis, has been found to plays a pivotal role in multiple stages of the viral life cycle, including viral entry, genome replication, and the encapsulation and release of viral particles (Aliyari et al., 2022; Shiota et al., 2023; Villareal et al., 2015). These findings indicate that suppressing key metabolic enzymes or targeting fatty acids and their derivatives might be an effective strategy to block viral infection and treat viral diseases.

In this review, we focus on recent progress in investigating the molecular mechanism of FASN expression regulation, the role of FASN in viral growth and infectious diseases, and how FASN could possibly be developed as a promising target in anti-virus therapies. By understanding these biological and pathological processes mediated by FASN, more valuable insights would be used for preventing virus spreading and infectious disease progression.

## Structure and expression distribution of FASN

FASN, also known as oncogenic antigen-519 (OA-519), is a prognostic molecule found in breast cancer that correlated with poor prognosis (Kuhajda et al., 1994). FASN contains two basic types of architectures and exists in eukaryotes as well as in prokaryotes (Jenni et al., 2007, 2006; Lomakin et al., 2007; Maier et al., 2006; Smith and Tsai, 2007; White et al., 2005). In bacteria and plants, FASN (also referred to as type II FASN) is a dissociated system involved in a series of individual and monofunctional proteins, which catalyzes fatty acid synthesis (White et al., 2005). It was also discovered in mammalian mitochondria, and some enzymes in the catalytic steps are homologous to prokaryotic proteins (Miinalainen et al., 2003; Zhang et al., 2003). In contrast, type I FASN, a more efficient cellular multienzyme, is found in fungi and higher eukaryotes (Pappenberger et al., 2010). Fungal FASN forms a 2.6-MD α_6_β_6_ heterododecamer (Jenni et al., 2006), whereas the human and animal FASN is an α_2_ homodimer composed of a single 270-kD polypeptide chain (Pappenberger et al., 2010). Human *FASN* gene has been mapped to chromosome 17q25 with 43 exons, spanning approximately 20 kb. Here, we focus on the function of type I FASN, which is overexpressed in many types of viral replication (Liu et al., 2021; Nasheri et al., 2013; Shen et al., 2023; Zheng et al., 2022), and FASN inhibitors have demonstrated anti-virus activity (Chu et al., 2021; Heaton et al., 2010; Kapadia and Chisari, 2005; Liu et al., 2024; Martin-Acebes et al., 2011; Miyanari et al., 2007; Perera et al., 2012; Sagan et al., 2006; Su et al., 2002).

FASN, a cytoplasmic protein, is absent or expressed at a minimal level under normal physiological conditions except in liver, brain, lung, and adipose tissue (Menendez and Lupu, 2007; Semenkovich, 1997). Human, pig, rat, mouse, goat, bovine, goose, and chicken FASN cDNAs have been cloned (Amy et al., 1989; Jayakumar et al., 1995; Kameda and Goodridge, 1991; Maier et al., 2008; Paulauskis and Sul, 1989; Roy et al., 2005; Yuan et al., 1988; Zhu et al., 2014), and the and the amino acid sequence contains six different catalytic domains required for synthesis of fatty acid, and an acyl carrier protein (ACP) domain, making it a multi-catalytic enzyme complex (Menendez and Lupu, 2007). The organization of these six enzymatic domains from the N terminus to the C terminus is as follows: β-ketoacyl synthase (KS), malonyl/acetyltransferase (MAT), dehydrase (DH), enoyl reductase (ER), β-ketoacyl reductase (KR), and thioesterase (TE) (Smith, 1994). These domain sequences are essential for dimer formation and function of FASN (Chirala et al., 2001).

*De novo* fatty acid synthesis and fatty acid uptake are two pivotal pathways for maintaining cellular lipid homeostasis. *De novo* fatty acid synthesis refers to the process by which cells synthesize fatty acids using non-lipid precursors, primarily acetyl-CoA and malonyl-CoA. This process not only provides cells with membrane structural components and energy reserves but also plays a role in signal transduction, immune regulation, and disease progression (Zhou et al., 2025). FASN serves as the rate-limiting enzyme in this pathway, catalyzing the *de novo* production of long-chain fatty acids and influencing the utilization of polyunsaturated fatty acids (PUFAs). Fatty acid uptake is the mechanism through which cells acquire exogenous fatty acids from circulation, primarily mediated by membrane transporters such as cluster of differentiation 36 (CD36), fatty acid transport proteins (FATPs), and fatty acid-binding proteins (FABPs) (Samovski et al., 2023). This uptake process is essential for maintaining systemic metabolic homeostasis and cellular functions. There exists a dynamic complementary and competitive relationship between fatty acid uptake and *de novo* synthesis. For instance, impaired fatty acid uptake due to lipoprotein lipase (LPL) deficiency leads to a compensatory upregulation of *de novo* synthesis-related enzymes (FASN, SCD1, ELOVL6) in adipose tissue, resulting in the abundant production of palmitoleic acid and myristoleic acid (Bartelt et al., 2013). Aberrant activation or imbalance of these two pathways is a common hallmark of various diseases, including obesity, cancer, and viral infections.

High-resolution co-crystal structures of porcine FASN (up to 3.2 Å resolution) have been determined using a multi-crystal data collection strategy (Maier et al., 2008). In this model, FASN assembles into an intertwined X-shaped homodimer. Porcine FASN is highly homologous to the human FASN, which contains two centrally connected parts: lower condensing unit—KS and MAT domains; and the modifying unit—DH, pseudo-methyltransferase (ΨME), pseudo-ketoacyl reductase (ΨKR), ER, KR, ACP, and TE domains. In addition, higher-resolution information of the isolated domains that include human FASN KS-MAT didomain (2.1 Å) (Pappenberger et al., 2010), MAT (2.8 Å) (Bunkoczi et al., 2009), TE (2.3 Å) (Chakravarty et al., 2004; Pemble et al., 2007), have been obtained for designing and developing catalytic domain-specific FASN inhibitors. Furthermore, continued work on the current crystal system have provide an atomic model of the core modifying region (DH:ΨME:ΨKR: ER:KR domains) of human FASN produced via the TEV cleavage method (Hasan et al., 2023). While these structures provide the basis for understanding the relationship between the mammalian FASN and polyketide synthases, little is known regarding the substrate shuttling mechanisms of FASN in dynamic aspects. Structural data with FASN in capturing intermediate states and obtaining kinetic information of FASN variants would be desirable. It is also unclear about the conformational dynamics of the FASN protein-drug complexes at high resolution. Approaches including the use of single molecule fluorescence spectroscopy and electron cryo-microscopy (cryoEM) structure determination of isolating FASN functional regions may help delineate these issues.

## Regulation of FASN expression and localization

**Figure 2:**
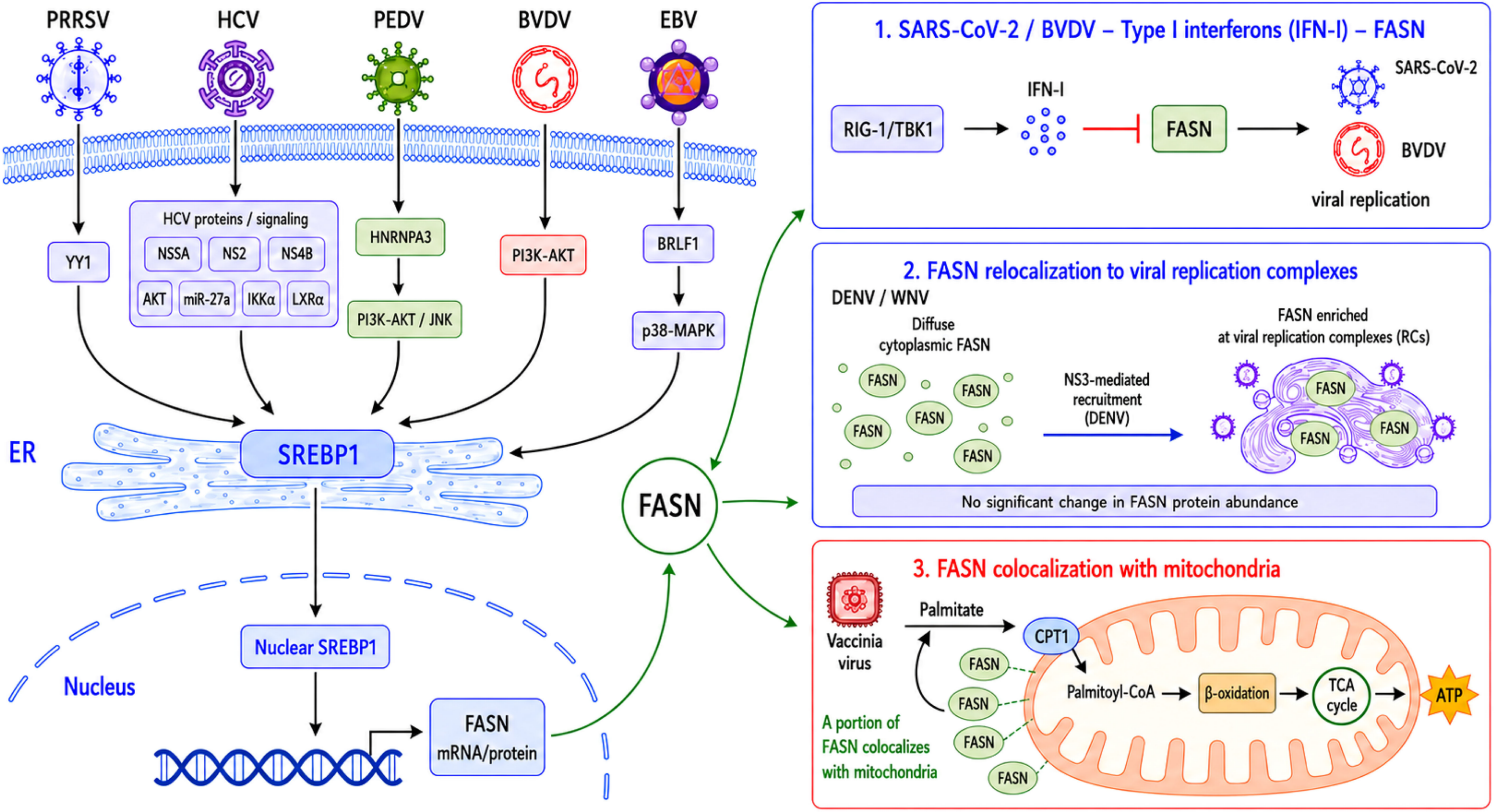
Molecular mechanism of FASN expression and localization regulation during viral infection. SREBP1, LXRα, and YY1 play a crucial role in regulating the expression of FASN by binding to its proximal promoter during HCV, PEDV and PRRSV infection. The PI3K-AKT and p38-MAPK signaling pathway also participate in regulating FASN during BVDV and EBV infection. DENV NS3 protein interacts with FASN and recruits FASN to sites of viral replication complexes (RCs). FASN co-localizes with mitochondria and facilitates the transport of palmitic acid, thereby promoting vaccinia virus proliferation. SREBP1, Sterol regulatory element-binding protein 1; LXRα, Liver X receptor α; YY1, Yin Yang 1.

FASN expression and activity are abnormally changed in some pathological conditions including cancer (Huang et al., 2024), obesity-related disorders (Matsukawa et al., 2023), nonalcoholic fatty liver disease (NAFLD) (Xu et al., 2024), inflammatory diseases (Balasubramanian et al., 2024), autoimmune diseases (Xiao et al., 2024a), and viral-related diseases (Liu et al., 2021; Zheng et al., 2024). In virus-infected cells or viral proteins over-expressing cells, the involvement of level of FASN mRNA and protein (Boodhoo et al., 2019; Li et al., 2004; Liu et al., 2021; Shi et al., 2024; Su et al., 2002; Yang et al., 2008; Yu et al., 2022; Zheng et al., 2022, 2024), subcellular localization of FASN (Heaton et al., 2010; Tang et al., 2014), and enzyme activity (Nasheri et al., 2013) has been found to be regulated (**Figure 2**). Because of FASN up-regulation, fatty acid biosynthesis (Heaton et al., 2010), *de novo* synthesized lipids (Heaton et al., 2010), triglyceride levels (Nasheri et al., 2013), and neutral lipid droplets (LDs) formation (Boodhoo et al., 2019; Liu et al., 2021; Shi et al., 2024; Tang et al., 2014; Zheng et al., 2022, 2024) were also elevated in accordance with FASN expression. These studies demonstrated efficient viral replication is so dependent on FASN and highlighted the therapeutic potential in the treatment of viral-related diseases by targeting the fatty acid biosynthetic pathway.

### Transcription factors

The regulation of FASN transcription is well studied and considered the crucial contributor to the increased FASN expression and activity in virus-infected cells (Li et al., 2004; Nasheri et al., 2013; Shi et al., 2024; Yu et al., 2022; Zheng et al., 2022, 2024). Sterol regulatory element-binding proteins (SREBPs), major nuclear transcription factors that take part in fatty acid synthesis, are important in activating FASN gene transcription via binding to the FASN promoter region (Deng et al., 2021; Garcia-Mediavilla et al., 2012; Horton et al., 2002; Jackel-Cram et al., 2007; Kim et al., 2007; Lim et al., 2021; Oem et al., 2008; Park et al., 2009). The SREBP family consists of three proteins: SREBP-1a (encoded by the *SREBF1* gene), SREBP-1c (produced from *SREBF1* gene splicing), and SREBP2 (produced from the *SREBF2* gene) (Eberle et al., 2004; Hua et al., 1995; Miserez et al., 1997). It has been reported that only SREBP-1a and SREBP-1c could mediate FASN transcription and fatty acid synthesis (Vanauberg et al., 2023; Xiao et al., 2024b). The involvement of SREBP-FASN axis in regulating viral replication was first reported in the development of hepatic steatosis by hepatitis C virus (HCV) infection, showing that FASN transcriptional activity up-regulation via HCV-3a and -1b core protein was mediated by SREBP1 in Huh-7 cells (Jackel-Cram et al., 2007). Similar observations have been found in Chang liver and Huh-7 cells, HCV core protein elevates the luciferase activity of FASN promoter by exogenous SREBP-1a and SREBP-1c (Kim et al., 2007). Additionally, the expression of HCV non-structural 2 (NS2) or NS4B protein in Huh-7 cells up-regulated FASN transcription in an SREBP-1-dependent manner (Oem et al., 2008; Park et al., 2009). A genome-wide RNA interference (RNAi) screen indicates that IκB kinase (IKK) α is requisite for productive HCV infection (Li et al., 2013). Furthermore, SREBP mediated up-regulation of FASN was abrogated by IKKα silencing using siRNA in Huh-7 cells (Li et al., 2013). Porcine epidemic diarrhea virus (PEDV) could hijack cellular lipid metabolism to benefit its replication, it was found that FASN activation was mediated primarily by the heterogeneous nuclear ribonucleoprotein A3 (HNRNPA3)/SREBP-1 signaling axis in PEDV-infected Marc-145 cells (Shi et al., 2024). In conclusion, all these findings suggest that SREBP-1 play an important role in modulating the expression of FASN during viral infections.

In an HCV *in vitro* model, the effects of HCV NS5A protein, core protein, and virus replication on transcription factor Liver X receptor (LXR) α-mediated FASN expression was examined by electrophoretic mobility shift assay (EMSA) and chromatin immunoprecipitation assay (ChIP assay) (Garcia-Mediavilla et al., 2012). It was clearly shown that HCV proteins and viral replication induced transcriptional activation of the FASN via increasing the binding of LXRα to LXR-response element (LXRE) promoter region of FASN (Garcia-Mediavilla et al., 2012). Curcumin, a phenolic compound, exhibits inhibitory ability against many viruses, including HBV (Rechtman et al., 2010), dengue virus (DENV) (Balasubramanian et al., 2019), porcine reproductive and respiratory syndrome (PRRSV) (Du et al., 2017), and classical swine fever virus (CSFV) (Gao et al., 2021). Subsequent studies of curcumin on CSFV infection showed that activating transcription factor 6 (ATF6) knockdown using siRNA significantly weakened the inhibitory effect of curcumin on FASN (Gao et al., 2021). In another study using fully differentiated porcine alveolar macrophages (PAMs) and piglets, transcription factor yin yang 1 (YY1) expression was found to increase after PRRSV infection and YY1 could inhibit PRRSV replication by reprogramming LD synthesis (Zheng et al., 2024). The luciferase reporter assay showed that YY1 directly regulated the promoter activity of FASN. Moreover, the authors found that YY1 affects PRRSV replication by manipulating FASN expression and FASN-mediated fatty acid synthesis (Zheng et al., 2024). These important findings suggest that FASN could be regulated by several different transcription factors and it may be developed as a target to inhibit viral infection.

### Proliferative signaling and FASN localization

The full activation of Epstein-Barr virus (EBV) early genes could be induced by the two EBV immediate-early (IE) proteins, BRLF1 and BZLF1 (Chevallier-Greco et al., 1986; Countryman and Miller, 1985; Feederle et al., 2000; Ragoczy et al., 1998; Westphal et al., 1999). The microarray analysis using an Affymetrix chip revealed that BRLF1 overexpression in telomerase-immortalized human primary keratinocytes (TIK) cells significantly increased the FASN mRNA level than BRLF1 non-expressing TIK cells (Li et al., 2004). Furthermore, Northern blot analysis showed that the p38 mitogen-activated protein kinase (p38 MAPK) signaling pathway activity is required for BRLF1-induced FASN activation in TIK cells (Li et al., 2004). During bovine viral diarrhea virus (BVDV) infection in CD8^+^T cells, the mRNA and protein expression levels of FASN were both promoted by RT-qPCR and Western blot analysis (Liu et al., 2024). Treatment of virus-infected cells with the PI3K inhibitor LY294002 confirmed that BVDV upregulates the expression of FASN by activating the PI3K/AKT signaling pathway (Liu et al., 2024). In another study, PI3K/AKT and JNK pathway, not MAPK pathway, mediated the effect of PEDV infection on FASN activation using Western blot analysis in Marc-145 cells (Shi et al., 2024). Although FASN is potentially under the aforementioned signaling pathways regulation, it is unclear if FASN contributes to and mediates the role of these pathways in different types of viral infections. It is also noteworthy that whether the MAPK pathway regulation of FASN in a virus type-dependent manner deserves to be investigated.

In addition to the above FASN mRNA level or protein expression involved in regulating viral replication, the subcellular localization of FASN have also been found to associate with viral infection (Greseth and Traktman, 2014; Heaton et al., 2010; Martin-Acebes et al., 2011; Nasheri et al., 2013; Perera et al., 2012; Tang et al., 2014). Although the protein abundance of FASN do not change significantly during DENV infection, its subcellular localization changes from the cytoplasm to sites of viral replication complexes (RCs) formation (Heaton et al., 2010). Furthermore, it was clearly demonstrated that DENV NS3 protein directly stimulates FASN activity without significant alterations in protein levels, and recruits FASN to sites of DENV replication via a specific interaction between NS3 and FASN (Heaton et al., 2010). Consistently, the association of FASN with the viral replication complex was observed during West Nile virus (WNV) infection, with no changes in the relative protein levels of FASN (Martin-Acebes et al., 2011). Although it remains unclear whether vaccinia virus infection influences the protein expression of FASN, a portion of FASN co-localizes with mitochondria and is thought to potentially facilitate the transport of palmitic acid, thereby promoting viral proliferation (Greseth and Traktman, 2014). Clearly, most studies as discussed above were at cellular level, so more *in vivo* animal models should be developed and used to demonstrate the regulation of FASN expression and activity in virus-related disease progression.

## Biological functions of FASN in viral infection

**Figure 3:**
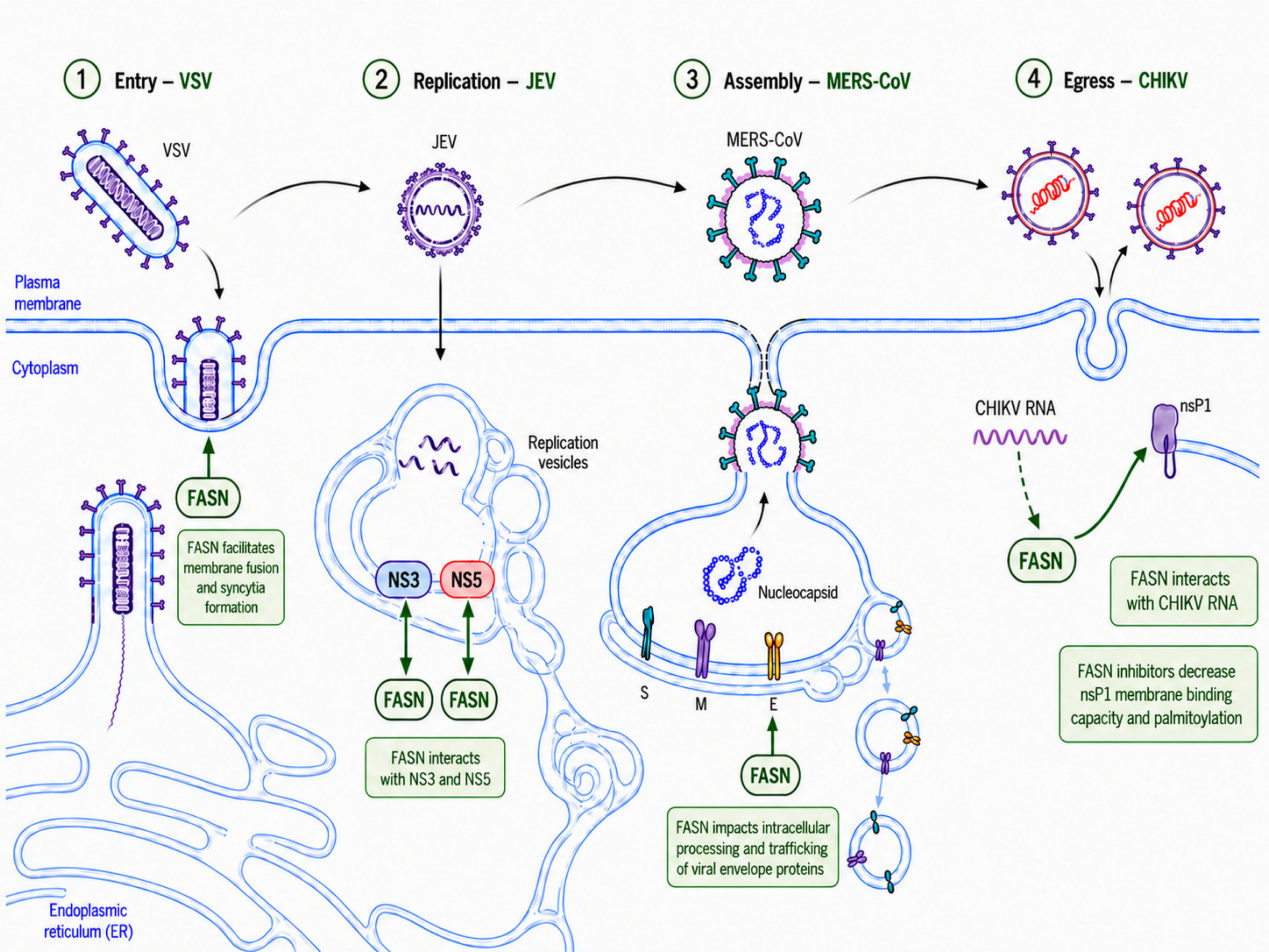
FASN’s roles across different stages of the viral life cycle. FASN facilitates membrane fusion and syncytia formation during VSV entry. JEV NS3 and NS5 interacts with FASN during viral replication. FASN impacts intracellular processing and trafficking of viral envelope proteins during MERS-CoV assembly. FASN increases CHIKV nsP1 membrane binding capacity and palmitoylation.

Viral diseases continue to pose a considerable threat to public health, with high morbidity and mortality rates on a global scale. It has been confirmed that FASN is involved in regulating the replication of various viruses via affecting post-translational modification of viral proteins, host immune response, and metabolic reprogramming (Albano et al., 2025; Aliyari et al., 2025; Bai et al., 2025; Li et al., 2025b; Ma et al., 2025b; Sornprasert et al., 2025; Soultsioti et al., 2025) (**Tables 1**–**3**). Several metabolic pathways, including fatty acid biosynthesis, lipid droplet formation, and fatty acid oxidation, directly or indirectly mediated by FASN, contribute to virus entry, replication, assembly, and egress (**Figure 3**). This section focuses on the FASN and its role in enhancing viral proliferation.

**Table 1:** Summary of the relationship between zoonotic virus replication and FASN.

Virus, type, genetic material	Genus and family	FASN inhibition by compounds and genetic approach	Inhibition stage and mechanism (ref.)
Dengue virus (DENV), Enveloped, RNA	*Flavivirus*, *Flaviviridae*	C75, cerulenin, orlistat, triclosan, lapatinib	Viral genome replication (Heaton et al., 2010; Sornprasert et al., 2025), lapatinib exhibits a potential direct-acting antiviral effect via NS5 (Sornprasert et al., 2025)
Zika virus (ZIKV), Enveloped, RNA	*Flavivirus*, *Flaviviridae*	Orlistat, lapatinib	Viral genome replication, lapatinib exhibits a potential direct-acting antiviral effect via NS5 (Sornprasert et al., 2025)
West Nile virus (WNV), Enveloped, RNA	*Flavivirus*, *Flaviviridae*	C75, cerulenin	Viral genome replication, FASN colocalizes with WNV replication complex (Martin-Acebes et al., 2011)
Japanese encephalitis virus (JEV), Enveloped, RNA	*Flavivirus*, *Flaviviridae*	Orlistat	Viral genome replication (Hitakarun et al., 2020), FASN interacts with NS3 and NS5 of JEV (Sornprasert et al., 2025)
Usutuvirus (USUV), Enveloped, RNA	*Flavivirus*, *Flaviviridae*	C75, cerulenin	Viral genome replication, FASN colocalizes with WNV replication complex (Martin-Acebes et al., 2011)
Chikungunya virus (CHIKV), Enveloped, RNA	*Alphavirus*, *Togaviridae*	C75, cerulenin, orlistat, FASN siRNA	Viral genome replication and release, FASN interacts with CHIKV RNA, FASN inhibitors decrease nsP1 membrane binding capacity and palmitoylation (Zhang et al., 2019; Bakhache et al., 2019)
Semliki Forest virus (SFV), Enveloped, RNA	*Alphavirus*, *Togaviridae*	Cerulenin	Viral genome replication (Royle et al., 2017)
Rift Valley Fever Virus (RVFV), Enveloped, RNA	*Phlebovirus, Phleboviridae*	C75, cerulenin	Viral genome replication (Moser et al., 2012)
Influenza A, Enveloped, RNA	*Alphainfluenzavirus*, *Orthomyxoviridae*	C75	The late stage of viral replication, C75 obstructs viral envelopment or lipid modification of viral proteins (Munger et al., 2008)
Rotavirus (RV), non-enveloped, RNA	*Rotavirus*, *Reoviridae*	C75, FASN siRNA	Viral assembly and release (Gaunt et al., 2013)
Vesicular stomatitis virus (VSV), Enveloped, RNA	*Vesiculovirus*, *Rhabdoviridae*	C75, cerulenin, FASN siRNA, pCMV-FASN	Viral entry, FASN facilitates membrane fusion and syncytia formation (Aliyari et al., 2022)
Middle East respiratory syndrome coronavirus (MERS-CoV), Enveloped, RNA	*Betacoronavirus*, *Coronaviridae*	TVB-2640	Viral assembly and release, FASN impacts intracellular processing and trafficking of viral envelope proteins (Soultsioti et al., 2025)
Monkeypox virus (MPXV), Enveloped, DNA	*Orthopoxvirus*, *Poxviridae*	C75, cerulenin, TVB‐3166, FASN knockout, FASN overexpression	Viral early infection (Aliyari et al., 2025)

**Table 2:** Summary of the relationship between human virus replication and FASN.

Virus, type, genetic material	Genus and family	FASN inhibition by compounds and genetic approach	Inhibition stage and mechanism (ref.)
Coxsackievirus B3 (CVB3), non-enveloped, RNA	*Enterovirus*, *Picornaviridae*	C75, cerulenin, orlistat, amentoflavone	Viral genome replication (Rassmann et al., 2007)
Rhinovirus, non-enveloped, RNA	*Enterovirus*, *Picornaviridae*	C75	Viral genome replication (Nguyen et al., 2018)
Enterovirus A71 (EV-A71), non-enveloped, RNA	*Enterovirus*, *Picornaviridae*	C75	Viral genome replication (Yang et al., 2022)
Astrovirus, non-enveloped, RNA	*Mammastrovirus*, *Astroviridae*	FASN siRNA	Viral genome replication (Murillo et al., 2015)
Mayaro virus (MAYV), Enveloped, RNA	*Alphavirus*, *Togaviridae*	Cerulenin, orlistat	Viral genome replication (Bakhache et al., 2019)
Respiratory syncytial virus (RSV), Enveloped, RNA	*Orthopneumovirus*, *Pneumoviridae*	TVB-3166	Viral genome replication, viral particle formation and infectivity of released viral particles (Ohol et al., 2015)
Severe acute respiratory syndrome coronavirus 2 (SARS CoV-2), Enveloped, RNA	*Betacoronavirus*, *Coronaviridae*	C75, EGCG, cerulenin, orlistat, TVB-2640, TVB-3166, TVB-3664, FASN siRNA, FASN knockout, pCMV-FASN	Viral genome replication (Aliyari et al., 2022)
Human Immunodeficiency Virus type 1 (HIV-1), Enveloped, RNA	*Lentivirus*, *Retroviridae*	C75, Fasnall	The late stage of viral replication (Kulkarni et al., 2017)
Hepatitis C virus (HCV), Enveloped, RNA	*Hepacivirus*, *Flaviviridae*	C75, cerulenin, orlistat, FASN siRNA, FASN shRNA	Viral genome replication (Nasheri et al., 2013; Huang et al., 2013), FASN enhances HCV NS5B RdRp activity by interacting with NS5B (Huang et al., 2013)
Kaposi’s Sarcoma-associated herpesvirus (KSHV), enveloped, DNA	*Rhadinovirus*, *Herpesviridae*	C75	Viral genome replication, C75 induces apoptotic death in infected cells (Delgado et al., 2012)
Human cytomegalovirus (HCMV), Enveloped, DNA	*Herpesviruses*, *Herpesviridae*	C75	The late stage of viral replication (Munger et al., 2008)
Epstein-Barr virus (EBV), Enveloped, DNA	*Lymphocytovirus*, *Herpesviridae*	C75	Viral genome replication (Li et al., 2004)
Herpes simplex virus type 1 (HSV-1), Enveloped, DNA	*Simplexvirus*, *Herpesviridae*	C75, CMS121, FASN shRNA	Viral assembly and envelope integrity (Albano et al., 2025), FASN inhibitors disrupt viral surface structure and virion morphology
Herpes simplex virus type 2 (HSV-2), Enveloped, DNA	*Simplexvirus*, *Herpesviridae*	UCM05	Viral entry, genome replication, envelope integrity, UCM05 directly binds to the HSV-2 glycoproteins gB and gD (Li et al., 2025b)
Hepatitis B virus (HBV), Enveloped, DNA	*Orthohepadnavirus*,*Hepadnaviridae*	GSK1995010, FASN siRNA	Viral assembly and release (Okamura et al., 2016)
Vaccinia virus, Enveloped, DNA	*Orthopoxvirus*, *Poxviridae*	C75	Viral assembly and release (Greseth and Traktman, 2014)

**Table 3:** Summary of the relationship between animal virus replication and FASN.

Virus, type, genetic material	Genus and family	FASN inhibition by compounds and genetic approach	Inhibition stage and mechanism (ref.)
Red-spotted grouper nervous necrosis virus (RGNNV), non-enveloped, RNA	*Betanodavirus*, *Nodaviridae*	C75, FASN siRNA	Viral entry and genome replication, FASN co-localizes with the CP protein (Huang et al., 2020)
Bovine viral diarrhea virus (BVDV), Enveloped, RNA	*Pestivirus*, *Flaviviridae*	C75	Viral adhesion, invasion, and genome replication, C75 activates the RIG-1/MDA-5-dependent IFN response (Liu et al., 2025b)
Classical swine fever virus (CSFV), Enveloped, RNA	*Pestivirus*, *Flaviviridae*	C75, FASN siRNA	The late stage of viral replication, FASN is recruited to CSFV replication sites in the ER and interacts with NS4B (Liu et al., 2021)
Porcine reproductive and respiratory syndrome virus (PRRSV) Enveloped, RNA	*Betaarterivirus*, *Arteriviridae*	EGCG, FASN siRNA	Viral genome replication and assembly (Yu et al., 2022)
Porcine epidemic diarrhea virus (PEDV), Enveloped, RNA	*Alphacoronavirus*, *Coronaviridae*	Palmitic acid	Viral genome replication (Mao et al., 2022)
Autographa californica multiple nucleopolyhedrovirus (AcMNPV), Enveloped, DNA	*Alphabaculovirus*, *Baculoviridae*	C75	Viral genome replication (Li et al., 2018)
Marek’s disease virus (MDV), Enveloped, DNA	*Mardivirus*, *Herpesviridae*	C75	Viral genome replication (Boodhoo et al., 2019)
Bovine alpha herpesvirus 1 (BoAHV-1), Enveloped, DNA	*Varicellovirus*, *Herpesviridae*	Cerulenin, FASN siRNA	Viral genome replication, FASN is localized in the Golgi apparatus and promotes the trafficking of virions out of the Golgi (Ma et al., 2025b)
Singapore grouper iridovirus (SGIV), Enveloped, DNA	*Ranavirus*, *Iridoviridae*	C75, FASN siRNA	Viral entry and genome replication, C75 increases the IFN immune and inflammatory Response (Zheng et al., 2022)

### Post-translational modification of viral proteins

Post-translational modifications of viral proteins play a crucial role in the viral life cycle and host-virus interactions. These modifications regulate the functions of viral proteins, influence viral replication and assembly, and facilitate adaptation to the host cellular environment (Loperena Gonzalez et al., 2025; Soultsioti et al., 2025; Zhang et al., 2019). The inhibition of ACC by TOFA or FASN by TVB-2640 significantly disrupts Middle East respiratory syndrome coronavirus (MERS-CoV) assembly and release via impacting intracellular processing and trafficking of viral envelope proteins in both Huh7 and MRC5 cells (Soultsioti et al., 2025). Western blot analysis of the structural proteins S, E, M, and N of MERS-CoV following TOFA treatment revealed a reduction in the trafficking of the S protein to the trans-Golgi network. Additionally, the migration rate of the E protein was consistently slightly faster, while the M protein exhibited an increased molecular weight, indicating alterations in the post-translational modifications of the viral proteins (Soultsioti et al., 2025). The pharmacological inhibitor Fasnall, along with the genetic knockout approach utilizing siRNA, effectively blocks a late stage of human immunodeficiency virus type 1 (HIV-1) replication in both HeLa-derived TZM-bl cells and primary PBMCs (Kulkarni et al., 2017). These results indicate that FASN activity is essential for efficient HIV replication. The potential mechanisms by which FASN promotes HIV replication may include the provision of fatty acids necessary for ATP production and energy homeostasis, as well as the generation of palmitate or myristate for the post-translational modification of the HIV-1 Gag protein. However, the specific mechanisms by which MERS-CoV and HIV-1 utilizes *de novo* synthesized fatty acids for the post-translational modification of viral protein and subsequent viral budding remain unclear. Investigating the cholesterol-rich lipid rafts within the plasma membrane, as well as palmitoylation and myristoylation of viral protein may help address some of these questions.

Influenza A, a zoonotic respiratory virus of major public health interest, is surrounded by an envelope composed of a lipid bilayer and membrane-spanning glycoproteins (Wachsmuth-Melm et al., 2025; Zhang et al., 2025). The inhibitors of fatty acid biosynthesis, like the acetyl coenzyme A carboxylase (ACC) inhibitor TOFA and the FASN inhibitor C75, impair influenza A infection, possibly by obstructing viral envelopment or lipid modification of viral proteins during the late stage of viral replication (Munger et al., 2008). Using siRNA and pharmacological inhibitors such as cerulenin and Orlistat, it was demonstrated that FASN expression and enzymatic activity are essential for CHIKV post-entry replication in both CHIKV reporter transreplication system and CHIKV-infected HEK293T cells (Bakhache et al., 2019). Furthermore, the decreased membrane binding capacity of CHIKV nsP1 was confirmed in HEK293T cells treated with cerulenin or Orlistat by confocal microscopy and the separation of cell membrane and cytoplasm (Bakhache et al., 2019). It suggested FASN is a proviral factor for CHIKV. Palmitoylation is widely recognized for its role in enhancing the infection of various alphaviruses by increasing host protein hydrophobicity and facilitating the trafficking of viral proteins to cellular membranes (Ahola et al., 2000; Li et al., 2025c; Zhang et al., 2021). Except for alteration of CHIKV nsP1 plasma membrane localization by FASN inhibitors, its palmitoylation is also regulated by FASN, and zinc finger DHHC domain containing palmitoyltransferases (ZDHHCs) such as ZDHHC2 and ZDHHC19 during viral replication (Zhang et al., 2019). Although the above findings indicate that FASN plays a crucial role in CHIKV replication by catalyzing the synthesis of palmitic acid, it remains unclear whether FASN also contributes to and mediates the palmitoylation of other CHIKV proteins, as well as host proteins involved in replication. More work should be conducted to further dissect the potential possibilities.

### Host immune response and cell membrane remodeling

Viruses can evade recognition and clearance by the host immune system by suppressing innate immunity and the functions of immune cells (Li et al., 2021; Ming et al., 2026). Type I interferons (IFN-I) serve as the crucial mediators that exert broad−spectrum antiviral activity. The *de novo* fatty acid synthesis catalyzed by FASN and its metabolic byproducts regulate viral replication by modulating the IFN-I signaling pathway (Liu et al., 2025b, 2024). UCM05, a potent inhibitor of FASN, dramatically suppresses HSV-2 at various stages of viral infection, including the inactivation of extracellular virions, virus entry, and intracellular replication (Li et al., 2025b). Furthermore, it enhanced the production of IFN-β and interferon-stimulated genes (ISGs), such as ISG15, ISG54, IFIT1, and IFIT3 (Li et al., 2025b). Numerous studies have indicated that targeting FASN may serve as a promising antiviral strategy for COVID-19 treatment (Aliyari et al., 2022; Chu et al., 2021; Suroengrit et al., 2024; Tanner and Alfieri, 2021; Williams et al., 2021). Although the cellular inhibition of FASN via C75 did not lead to an increase in pro-inflammatory or IFN-I cytokines, the downregulation of FASN by IFN-I as a defense mechanism may represent an effective strategy for combating a wide variety of enveloped viruses, including SARS-CoV-2 and its variants of concern (Aliyari et al., 2022). In addition to FASN, this study further reveals that IFN-I also suppresses the expression of other lipid metabolism-related genes, such as Acetyl-CoA carboxylase alpha (ACACA), which regulates *de novo* fatty acid biosynthesis, and Acyl-CoA synthetase long-chain family member 3 (ACSL3), which mediates diacylglycerol synthesis. Using the K18-hACE2 transgenic mouse *in vivo* model, both orlistat and TVB-2640 treatments administered via injection resulted in lower SARS-CoV-2 viral titers in lung tissues, inhibited lung inflammation, and prolonged survival in mice (Chu et al., 2021). These results demonstrate that FASN can influence viral proliferation by either inhibiting the IFN-I pathway or being downregulated through this pathway. However, the crosstalk mechanism between innate immune pathways and host metabolism, as well as the potential involvement of viral proteins in this process, remains unclear.

Bovine viral diarrhea virus (BVDV) has resulted in significant economic losses for the global cattle industry. It has been documented that the reciprocal regulation between FASN and IFN-I pathway plays a critical role throughout the entire replication cycle of BVDV (Liu et al., 2025b, 2024). C75 exhibited inhibitory effects at various stages of BVDV infection, including adhesion, invasion, and replication (Liu et al., 2025b). Mechanistically, RIG-1 dependent IFN-I responses act as a primary mediator in the FASN-driven modulation of BVDV propagation in both CD8^+^T cells and BT cells (Liu et al., 2025b, 2024). Moreover, C75 significantly enhanced the expression of ISGs in the mouse spleen through the activation of the RIG-I/TBK-1 signaling pathway (Liu et al., 2025b). In another study investigating the regulation of interferons by lipid metabolism, it was found that HCV infection induces lipid accumulation in host cells and downregulates the expression of the IFN-I receptor subunit IFNAR1 (Gunduz et al., 2012). This downregulation triggers dysfunction of the Jak-Stat signaling pathway, ultimately attenuating the antiviral immune response. Consistently, CSFV-induced accumulation of free fatty acids results in impaired antiviral responses mediated by IFN-I signaling due to the down-regulation of RIG-I-like receptors (RLRs) and their associated signaling molecules (Ma et al., 2019).

Targeted inhibition of FASN can consistently suppresses Singapore grouper iridovirus (SGIV) replication in grouper spleen (GS) cells. Notably, during this process, FASN may play crucial roles in SGIV infection via enhancing virus entry and downregulating the host immune response (Zheng et al., 2022). Given the essential role of FASN and its mediation of fatty acid synthesis in host innate immunity, further studies are required to elucidate the mechanisms through which FASN inhibition triggers the host immune response. These mechanisms include the interplay between FASN and the IFN signaling pathway, as well as the post-translational modifications of pathway-related molecules by palmitate or myristate, which ultimately facilitate the clearance of viral infections.

In studies concerning the adaptation of the adaptive immune system to lipid metabolism, it has been demonstrated that influenza virus infection elevates the expression of antiviral genes by downregulating lipid metabolism-associated genes in murine CD4^+^ T cells (Kanno et al., 2021). Mechanistically, the regulation of T-cell antiviral responses induced by the cGAS-STING signaling axis is critically influenced by the restriction of triacylglycerol-containing monounsaturated fatty acids (MUFAs) (Kanno et al., 2021). This is achieved either by inhibiting the fatty acid synthesis pathway or by knocking out the stearoyl-CoA desaturase (SCD) gene, *Scd2*. These findings underscore the importance of clarifying the role of MUFAs in enhancing basal ISGs expression and in shaping a lipid microenvironment that supports the defensive functions of host CD4^+^ T cell subsets. Furthermore, it is essential to conduct further in-depth investigations into whether MUFAs can regulate the IFN-I response in immune cells during other viral infections.

The homeostasis of cell membrane components serves as a critical regulatory factor in viral replication. Viruses utilize various mechanisms, including the alteration of cell membrane integrity, induction of membrane remodeling, and hijacking of cellular lipid metabolism, to facilitate successful invasion, replication, and propagation (Fu et al., 2026; Wang et al., 2025). FASN inhibition by TVB-3166 treatment significantly reduces infectious progeny of multiple RSV strains by altering the host cell membrane composition, which is crucial for RSV assembly or replication. This effect has been observed both in an *in vitro* A549 cell model and in an *in vivo* mouse model (Ohol et al., 2015). The addition of exogenous palmitate to TVB-3166-treated cells restores RSV production by increasing the phosphorylation levels and activity of FASN in the cell membrane fractions. Although FASN inhibition reduces viral protein expression, it remains unclear whether this effect is due to altered protein translation or enhanced protein degradation. Furthermore, it is still uncertain whether lipid rafts that can associate with viral proteins play a role in regulating viral replication under these conditions.

### Lipid droplet biogenesis and fatty acid synthesis

LDs serve as scaffolds for viral RNA replication and facilitate the assembly and maturation of viral particles through their interaction with viral capsids (Laufman et al., 2019). The biogenesis of LDs increases in response to elevated fatty acid levels, and they have been observed to co-localize with viroplasms during rotavirus (RV) infection (Cheung et al., 2010). The combined treatment with isoproterenol and isobutylmethylxanthine (IBMX), two chemical compounds known to interfere with LD formation, resulted in a decreased number of viroplasms and a reduced production of infectious progeny viruses (Cheung et al., 2010). In another study, it was demonstrated that Ras-related proteins in brain (Rab)18 could recruit FASN to interact with DENV NS3 protein and to the site of DENV replication, LDs (Tang et al., 2014). This recruitment facilitates viral RNA replication and virion assembly, processes that require substantial amounts of lipids (Tang et al., 2014). Furthermore, the independent interaction of FASN with NS3 of DENV, ZIKV, and JEV was confirmed by co-immunoprecipitation (Co-IP) in HEK293T/17 cells during natural infection (Sornprasert et al., 2025). Notably, RV, a non-enveloped enteric virus, promotes LD synthesis by increasing fatty acid levels and recruit LDs to its replication complexes, thereby forming a structural framework for viral assembly.

Regarding classical swine fever (CSF), FASN activity and LDs accumulation are prerequisite for CSFV replication (Bai et al., 2025; Gao et al., 2021; Liu et al., 2021). *In vitro* experimental results showed that FASN knockdown or treatment with C75 significantly impairs the late stage of CSFV propagation by reducing the formation of LDs (Liu et al., 2021). Enterovirus A71 (EV-A71) replication is intricately linked to the formation of LDs. The RCs of EV-A71 facilitate the recruitment of host LDs to their periphery via membrane contact sites (Yang et al., 2022). Treatment with C75 inhibits lipid droplet formation, consequently restricting the availability of fatty acids for β-oxidation and ultimately impeding viral replication.

Fatty acids and the genes associated with their synthesis play a crucial role in the replication of viruses and the progression of virus-associated diseases (Deshmukh et al., 2025; Li et al., 2025d; Trubl et al., 2025). Fatty acids exist in various forms, derived from palmitate through mechanisms such as chain shortening (e.g., myristate [C14:0]), chain elongation (e.g., stearate [C18:0]), and desaturation (e.g., oleate [C18:1]). These fatty acids play pivotal roles in regulating complex lipid biosynthesis, including phospholipids, sphingolipids, and triacylglycerols, as well as in membrane fusion and energy production (Cockcroft, 2021). Upon the infection of Huh-7.5 hepatocellular carcinoma cells with hepatitis A virus (HAV), untargeted high-resolution liquid chromatography coupled with tandem mass spectrometry (LC-MS/MS) was utilized to analyze alterations in cellular lipid profiles. The results indicated that HAV infection significantly increased the relative abundance of intracellular lipids, including phosphatidylcholine, cholesterol esters, and triglycerides. Additionally, the proportion of lipids containing very-long-chain fatty acids was markedly elevated. Conversely, the levels of phospholipids containing polyunsaturated fatty acids with multiple double bonds were found to be decreased (Shiota et al., 2023). Furthermore, genome-wide CRISPR screening and transcriptomic analyses reveal that ACC1, very-long-chain 3-oxoacyl-CoA reductase (17β-HSD12), and the very-long-chain fatty acid (VLCFA) elongases ELOVL4 and ELOVL7 play significant roles in modulating viral replication through the regulation of lipid metabolism (Shiota et al., 2023). Inhibition of FASN activity decreases the supply of fatty acids and alters their composition, thereby impairing the synthesis of phospholipids (Yin et al., 2025), including phosphatidylcholine (PC), phosphatidylethanolamine (PE), phosphatidylserine (PS), and phosphatidylinositol (PI). This phospholipid synthesis is essential for the formation of RCs (Zhang et al., 2018).

In a study investigating the effects of human cytomegalovirus (HCMV) infection on host metabolism, it was observed that saturated very-long-chain fatty acids are upregulated during viral replication (Purdy et al., 2015). This upregulation supports the formation of the viral envelope and the production of infectious progeny virions. Screening of the fatty acid elongase family indicated that these fatty acids are predominantly produced by ELOVL7 during viral infection. Furthermore, HCMV protein pUL38 modulates mTOR-SREBP1 activity, which subsequently induces the ELOVL7-mediated synthesis of saturated very-long-chain fatty acids (VLCFAs), ultimately facilitating efficient viral replication (Purdy et al., 2015). Given the complexity of viral envelope formation, further studies are required to elucidate how long-chain fatty acids interact with viral proteins to establish a lipid microenvironment conducive to viral replication.

Multi-omics analyses and molecular biology techniques have demonstrated that the elevated expression of FASN is tightly linked to enhanced replication of various enveloped and non-enveloped RNA viruses (Bai et al., 2025; Huang et al., 2020; Liu et al., 2025b, 2024; Shi et al., 2024; Zheng et al., 2024). Based on metabolomic approaches concerning viral infections, recent studies have highlighted the novel mechanisms by which viruses utilize fatty acid biosynthesis to replicate efficiently within their hosts, a process that is also linked to cancer development. For example, metabolomic analysis of KSHV-infected tert-immortalized microvascular endothelial (TIME) cells indicated that fatty acid synthesis is essential for KSHV infection and ultimately KS tumors (Delgado et al., 2012). Subsequent studies demonstrated that treatment with C75 effectively inhibits KSHV latent infection by inducing apoptotic death in infected cells (Delgado et al., 2012). EBV, a ubiquitous human herpesvirus, has been associated with various malignancies, including Hodgkin’s lymphoma, diffuse large B-cell lymphoma, nasopharyngeal carcinoma, and gastric carcinoma (Liu et al., 2025a; Stewart and Damania, 2025). In an *in vitro* model, the engagement of the B-cell receptor in Akata cells, along with C75 treatment, resulted in a reduction in the expression of three lytic proteins of EBV: BZLF1, BRLF1, and BMRF1 (Li et al., 2004). Despite disrupting FASN activity may represent an effective strategy for suppressing lytic EBV replication, current EBV-specific treatments and non-toxic strategies in patients remain predominantly in the research phase. Thus, an in-depth investigation into the fatty acid synthesis pathway regulated by EBV is crucial for developing novel targeted therapies against EBV-associated malignancies.

Both TOFA and cerulenin treatments exhibited significant antiviral effects against SFV via targeting fatty acid synthesis in A549 cells infected with a recombinant strain of SFV expressing cleavable firefly luciferase (Royle et al., 2017). Furthermore, fatty acid synthesis, which is indirectly regulated by liver kinase B1 (LKB1) and AMP-activated protein kinase (AMPK), plays a crucial role in RVFV infection (Moser et al., 2012). Consistently, WNV, USUV, and AcMNPV, have been demonstrated to rely on fatty acid synthesis for their replication, specifically viral RNA synthesis, as revealed upon C75 treatment (Martin-Acebes et al., 2011). Coxsackievirus B3 (CVB3) and rhinovirus are small, non-enveloped RNA viruses (Li et al., 2025a; Liu et al., 2025c; Ma et al., 2025a). It has been demonstrated that the replication of these viruses relies on functional FASN effects and fatty acid synthesis, as evidenced by treatments with FASN inhibitors, including C75, cerulenin, amentoflavone, and orlistat (Nguyen et al., 2018; Rassmann et al., 2007; Wilsky et al., 2012; Yang et al., 2022). Silencing FASN using short hairpin RNA (shRNA) or applying C75 substantially diminishes the production of HSV-1, HBV, and vaccinia virus infectious progeny without affecting viral protein expression or genome replication (Albano et al., 2025; Greseth and Traktman, 2014; Okamura et al., 2016). Moreover, genetic manipulation strategies, such as FASN knockdown and overexpression, can either promote or inhibit the replication of various viruses, including vesicular stomatitis virus (VSV) (Aliyari et al., 2022), Nipah virus (NiV) (Aliyari et al., 2022), and human astrovirus (Murillo et al., 2015). Although the aforementioned studies have confirmed that FASN influences viral replication through various mechanisms, it remains unclear whether FASN exerts analogous effects at different stages of the viral replication cycle and whether it regulates the interactions between viral proteins and host cell membranes.

Using TOFMS combined with multivariate statistical analysis, it was found that red-spotted grouper nervous necrosis virus (RGNNV), a non-enveloped RNA virus, induced an increase in fatty acid biosynthesis and altered the distribution of FASN, which is partially co-localized with the CP protein (Huang et al., 2020). Further, C75 treatment and FASN knockdown using siRNA both remarkably decreased RGNNV infection at different stages of the viral life cycle, including virus entry and replication (Huang et al., 2020). In addition, FASN-mediated fatty acid synthesis and lipid accumulation are also involved in regulating PRRSV and PEDV replication (Mao et al., 2022; Shi et al., 2024; Yu et al., 2022; Yuan et al., 2025; Zheng et al., 2024). PRRSV, a highly infectious RNA virus, poses a major threat to global swine production, resulting in substantial economic losses (Chen et al., 2025; Costantini and Donini, 2025; Li et al., 2017). It has been confirmed that FASN-mediated fatty acid synthesis plays a key role in the influence of YY1 on PRRSV replication. For instance, the silencing of FASN counteracted the enhancement of PRRSV replication induced by YY1 knockout (Zheng et al., 2024). SREBF1 downregulation using siRNA in Marc-145 cells impairs PEDV replication through affecting lipid synthesis and its downstream genes, FASN and ACC1 (Shi et al., 2024). In a study examining the therapeutic effects of quercetin on PEDV infection, it was confirmed that PEDV infection promotes the accumulation of LDs (Gong et al., 2024). Mechanistically, treatment with quercetin inhibits LDs accumulation by downregulating the NF-κB signaling pathway and reducing the levels of interleukin-1 beta (IL-1β), IL-8, and IL-6, thereby suppressing viral replication (Gong et al., 2024). In contrast, palmitic acid, a metabolite downstream of FASN in the fatty acid synthesis pathway, significantly represses PEDV proliferation in porcine small intestinal enteroids (2-D enteroids) and IPEC-J2 cells (Mao et al., 2022). These contradictory results may be attributed to variations in viral strains, viral cell tropism, the necessity of lipid metabolism, the utilization of cellular receptors, and the regulation of these receptors by lipid metabolism. The entry of PEDV into host cells is a rapid and intricate process. To date, no definitive functional receptor for PEDV has been identified; instead, viral entry is generally co-regulated by multiple cell surface molecules, including aminopeptidase N (APN), transferrin receptor 1 (TfR1), heparan sulfate, sialic acid, interferon-induced transmembrane proteins (IFITM1/2/3), and integrin αvβ3. Following the binding of PEDV virions to target cells, they are internalized in a manner that is dependent on dynamin and cholesterol. This internalization is primarily achieved through clathrin-mediated endocytosis (CME) and caveolae-mediated endocytosis (CavME). Both downregulating SREBF1 expression through siRNA and quercetin treatment inhibit PEDV infection primarily at the viral replication stage. These interventions likely exert their antiviral effects by modulating LDs accumulation and the formation of RCs. Conversely, treatment with palmitic acid in 2-D enteroids and IPEC-J2 cells may suppress PEDV proliferation by impairing viral entry and internalization. Further studies are necessary to elucidate how PEDV hijacks host lipid metabolism at various replication stages to support its propagation, as well as to determine whether lipid metabolic dysregulation interferes with viral entry.

Elevated cholesterol levels can promote the expression of genes associated with fatty acid synthesis and facilitate fatty acid accumulation (Xiao et al., 2025). The cholesterol-25-hydroxylase (CH25H) and its enzymatic product, 25-hydroxycholesterol (25HC), exhibit broad-spectrum antiviral activity properties by altering membrane fluidity and lipid raft architecture, inhibiting *de novo* fatty acid synthesis, and promoting fatty acid β-oxidation (Yang et al., 2026; Zang et al., 2020). For instance, CH25H can inhibit viral entry through its enzymatic product, 25HC, significantly reducing the replication of Newcastle disease virus (NDV) (Zhu et al., 2025). Mechanistically, CH25H interacts with the viral nucleoprotein (NP), leading to a downregulation of NP expression and a suppression of the viral ribonucleoprotein (RNP) complex activity (Zhu et al., 2025). Monkeypox virus (MPXV) infection results in the downregulation of FASN and the upregulation of CH25H (Aliyari et al., 2025). The detection of key molecules in the innate immune pathway indicates that IFN-I inversely regulates the expression of CH25H and FASN, consequently inhibiting the replication of MPXV. Furthermore, both 25HC and C75 treatments have demonstrated significant antiviral activity (Aliyari et al., 2025). Although the above studies have confirmed that CH25H and FASN are simultaneously involved in regulating viral proliferation and are modulated by IFN signaling, the regulatory mechanisms linking the innate immune system and host metabolic pathways during viral replication remain poorly understood. This gap represents a promising direction for further research. Additionally, whether viruses can evade innate immunity by regulating cholesterol synthesis and fatty acid metabolism, as well as how CH25H and FASN modulate the expression of viral proteins, warrants further in-depth investigation.

Both acute and latent infections with bovine alpha herpesvirus 1 (BoAHV-1) result in the downregulation of FASN protein expression in trigeminal ganglia (TG) neurons (Ma et al., 2025b). Similarly, FASN protein expression demonstrates a decreasing trend during productive BoAHV-1 infection in both MDBK and Neuro-2A cells. Knockdown of FASN using siRNAs leads to a decrease in the replication of BoAHV-1. This was assessed by detecting the protein expression of virion-associated proteins in Madin-Darby bovine kidney (MDBK) cells (Ma et al., 2025b). In terms of mechanism, a portion FASN is localized in the Golgi apparatus and promotes the trafficking of virions out of the Golgi, a process that is crucial for the completion of the BoAHV-1 replication cycle (Ma et al., 2025b). In the studies examining the alterations in FASN expression during viral replication, it has been observed that the majority of viral infections lead to an upregulation of FASN expression. In contrast, infections with MPXV and BoAHV-1 result in a reduction of FASN mRNA levels and protein expression, respectively. Notably, following BoAHV-1 infection, the level of FASN mRNA is significantly elevated. The discrepancies observed between mRNA and protein levels may be attributed to factors such as decreased mRNA stability, reduced translational efficiency, and protein degradation through the ubiquitin-proteasome pathway or the lysosomal degradation pathway. Although infection with MPXV and BoAHV-1 results in decreased expression of FASN, this enzyme still plays a proviral role in the replication of both viruses. The downregulation of FASN could signify a host stress response to viral infection. Analyzing how MPXV and BoAHV-1 reduce FASN expression requires examining factors such as abnormal transcription factor expression, activation of the ubiquitin–proteasome pathway, O-GlcNAc modification, and inhibition of the AMPK or mTOR signaling pathways.

## FASN as therapeutic target

Numerous studies have focused on the main viral proteins, particularly proteases, for drug screening to identify novel therapeutic agents for the treatment of virus-associated diseases (Baranovskiy et al., 2025; Cavina et al., 2025; Ni et al., 2025; Qiao et al., 2021; Xu et al., 2025; Zhang et al., 2020). High-throughput screening of clinical-stage or US Food and Drug Administration (FDA)-approved small molecules has been conducted to expedite the development of these drugs. However, progress in developing drugs that target viral proteins has been sluggish due to variability among different viruses, which encode distinct enzymes, as well as limitations in clinical efficacy. Based on viral replication is heavily reliant on the host’s metabolic system, FASN has been identified as a promising therapeutic target by metabolomic profiling and the screening of a short-hairpin RNA sublibrary that targets metabolic genes (Chu et al., 2021; Farley et al., 2025).

There is substantial evidence that inhibitors of FASN and FASN mediated fatty acid synthesis modulate the replication of various viruses and the progression of related diseases (Chu et al., 2021; Delgado et al., 2012; Li et al., 2025b; Yu et al., 2022; Zheng et al., 2024). For example, UCM05, a safe small molecule compound, has recently been identified as an effective FASN inhibitor, demonstrating concentration-dependent anti-HSV-2 activity both *in vitro* and *in vivo* models (Li et al., 2025b). The MTT assay was conducted to assess the cytotoxicity of UCM05 against four HSV-2 target cell lines: Vero, HeLa, D407, and Beas-2B. The half-maximal cytotoxic concentration (CC_50_) was found to range from 32.58 ± 0.49 µM to 88.88 ± 2.2 µM. Following the determination of the HSV-2 DNA copy number in infected cells, the half-maximal inhibitory concentration (IC_50_) of UCM05 was calculated to be between 1.57 ± 0.6 µM and 3.67 ± 0.24 µM. The therapeutic index (TI) consistently exceeded 20, underscoring the high specificity and safety profile of this inhibitor. However, in subsequent cytopathic effect assays and mouse therapeutic experiments, UCM05 demonstrated enhanced efficacy primarily at relatively higher concentrations. For instance, in HSV-2-infected Vero cells, higher concentrations of UCM05 (5 and 10 µM) exhibited significantly stronger inhibition of cytopathic effect (CPE) compared to the control group and cells treated with lower doses of UCM05 (1.25 µM and 2.5 µM). In the HSV-2-infected mouse model, the high-dose UCM05 group (30 mg/kg/day) displayed superior therapeutic outcomes in comparison to both the control and low-dose UCM05 group (15 mg/kg/day). Further mechanistic investigations indicated that UCM05 may enhance antiviral immunity by modulating IFN-I production. Given the essential role of FASN in maintaining physiological lipid homeostasis-particularly in the liver and central nervous system, as well as in immune responses, the systemic toxicity risks associated with FASN inhibitors merit serious attention. It is also crucial to investigate whether FASN inhibitors exert differential regulation on host immune responses under distinct physiological and pathological conditions.

The genetic approach to modifying lipid metabolism gene expression in animal models presents a promising alternative for antiviral therapy and the treatment of related diseases. Although animal models with FASN knockdown and overexpression have not yet been employed to explore treatments for viral infectious diseases, studies focusing on PRRSV have utilized piglets infected with a recombinant lentivirus expressing YY1 (Zheng et al., 2024). This approach weakens the fatty acid synthesis pathway by negatively regulating the expression of FASN.

In addition, natural products derived from plants have contributed significantly to treatment of various viral infectious diseases. For instance, epigallocatechin gallate (EGCG), a polyphenolic compound from green tea, inhibits the proliferation of PRRSV primarily during viral replication and assembly by disturbing lipid metabolism (Yu et al., 2022). Quercetin, a natural polyhydroxy flavonoid derived from rutin, exhibits potent antiviral activity against PEDV in African green monkey kidney (Vero) cells and piglets via inhibiting LD accumulation (Gong et al., 2024). Although EGCG and quercetin inhibit viral proliferation through distinct pathways, their underlying antiviral mechanisms remain to be fully elucidated. Key unresolved questions include whether these compounds target and disrupt the activity of critical cell surface molecules involved in viral entry, and whether they modulate FASN expression to influence the palmitoylation of viral proteins. Meanwhile, future research should also focus on their potential for clinical application and conduct relevant pharmacokinetic and pharmacodynamic analyses.

Notably, the role of FASN and cellular lipid metabolism in regulating the infection of PEDV is controversial and debatable depending on the model systems and PEDV strains used for each study. Investigations into the proliferation of PEDV strain CH/SXYL/2016 (GenBank: MF462814.1) via the HNRNPA3/SREBP-1 signaling axis (Shi et al., 2024), as well as the effects of quercetin on PEDV strain GDS01 (Genebank: KM089829.1) (Gong et al., 2024), have indicated a positive correlation between FASN expression, lipid accumulation, and viral replication. Conversely, in studies involving PEDV strain ZJ15XS0101 (GenBank: MK409657.1 and MK409659.1) in either enteroids or IPEC-J2 cells, palmitic acid treatment significantly reduced viral replication (Mao et al., 2022). Although lipid metabolism exerts either a positive or negative regulatory effect on PEDV proliferation, the precise molecular mechanisms in this process have not been fully elucidated. Furthermore, it is yet unknown if a similar regulatory pattern exists in animal infection models. Similarly, it remains to be determined whether different strains of other viruses exhibit analogous phenomena when influenced by FASN and lipid metabolism. Clearly, future studies are necessary to address these complex issues. The development of animal models and the use of diverse virus strains will be instrumental in resolving some of these questions.

Using the HEK293T-hACE2 cell model, the efficacy of 22 FASN inhibitors in controlling SARS-CoV-2 replication was tested through fluorescence microscopy analyses (Chu et al., 2021). Among these inhibitors, Orlistat, an anti-obesity drug approved by the US FDA, and TVB-2640, which is currently undergoing phase II clinical trials as an anticancer therapy, were highlighted. Both Orlistat and TVB-2640 exhibited significant inhibitory effects on SARS-CoV-2, with half maximal effective concentration (EC_50_) values of 390 nM and 4 nM, respectively. Furthermore, it was confirmed that both compounds effectively suppress SARS-CoV-2 infection across multiple cell culture models. Specifically, the effective antiviral concentrations were determined to be 20 μM for orlistat and 2 μM for TVB-2640 in human lung cell lines (NCI-H1355 and NCI-H1437), 80 μM for orlistat and 2 μM for TVB-2640 in human colon cell line (Caco-2), and 5 μM for orlistat and 1 μM for TVB-2640 in hACE2-overexpressing mouse embryonic fibroblasts (MEF-hACE2) (Chu et al., 2021). In the K18-hACE2 mouse model of SARS-CoV-2 infection, daily intraperitoneal administration of orlistat (8 mg/kg) or TVB-2640 (8 mg/kg) demonstrated significant *in vivo* anti-SARS-CoV-2 activity. This was primarily evidenced by the suppression of pulmonary inflammation, attenuation of disease progression, and improved survival rates among virus-infected mice (Chu et al., 2021). Although both orlistat and TVB-2640 exhibit favorable therapeutic efficacy and safety, it remains to be clarified whether these two inhibitors exert their antiviral effects solely by regulating FASN. The observation that palmitic acid only partially reverses the antiviral activity of orlistat suggests that orlistat possesses lipase-inhibiting activity in addition to suppressing FASN function. Further studies are necessary to elucidate the precise molecular mechanism by which orlistat acts throughout the viral life cycle.

## Future perspectives and conclusions

FASN is a macromolecular metabolic enzyme that plays a crucial role in the replication of zoonotic, human, and animal viruses. While a limited number of studies have reported that viral infection downregulates FASN expression (Aliyari et al., 2025; Ma et al., 2025b), the majority of research consistently demonstrates that FASN expression and enzyme activity are upregulated following viral infection and positively regulate viral proliferation (Boodhoo et al., 2019; Heaton et al., 2010; Liu et al., 2024, 2021; Nasheri et al., 2013; Soultsioti et al., 2025). Further studies are clearly needed to delineate the causes of these differences, with the aim of determining whether FASN plays distinct roles at various stages of viral infection. During viral infections, the regulation of FASN occurs through multiple mechanisms. These mechanisms include modulation by transcription factors such as SREBP-1, LXR, ATF6, and YY1, as well as involvement of the miR-27a, p38-MAPK, and PI3K/AKT signaling pathways, and alterations in subcellular localization. However, the interaction dynamics between the aforementioned FASN-regulatory factors and viral proteins remain largely unknown. Furthermore, it remains to be validated whether their regulatory effects on FASN are applicable to the replication of other viruses.

FASN can participate in the viral replication cycle through multiple regulatory mechanisms, including: (a) modulating the synthesis of viral proteins; (b) interacting with viral proteins and forming complexes; (c) mediating viral protein palmitoylation; (d) facilitating fusion between viral and cellular membranes and their trafficking across different cellular organelles; (e) inducing cellular apoptosis; and (f) triggering the host innate immune response. Given the critical role of FASN in the replication of various viruses, the available data suggest that FASN is a promising broad-spectrum antiviral target with significant application prospects. However, there remain considerable gaps in understanding the mechanisms of FASN’s action in virus-related diseases and the clinical applications of FASN inhibitors that require further investigation. Elucidating the role of FASN in modulating host immune responses and immune cell functions during viral infections will enhance our understanding of its underlying mechanisms of action. Expanding animal experiments and clinical trials is crucial for advancing the development and clinical implementation of FASN-targeted therapeutics. Therefore, more efforts are necessary to explore the possibility of establishing FASN as a therapeutic target for viral infections and virus-associated diseases.
